# Improvements in viral gene annotation using large language models and soft alignments

**DOI:** 10.1186/s12859-024-05779-6

**Published:** 2024-04-25

**Authors:** William L. Harrigan, Barbra D. Ferrell, K. Eric Wommack, Shawn W. Polson, Zachary D. Schreiber, Mahdi Belcaid

**Affiliations:** 1 Hawai’i Institute of Marine Biology, University of Hawai’i at Mānoa, Honolulu, HI 96822 USA; 2 Department of Plant & Soil Sciences, University of Delaware, Newark, DE 19713 USA; 3 Department of Computer and Information Sciences, University of Delaware, Newark, DE 19713 USA; 4 Department of Computer Science, University of Hawai’i at Mānoa, Honolulu, HI 96822 USA

**Keywords:** Large language models, Protein homology, Viruses, Alignments

## Abstract

**Background:**

The annotation of protein sequences in public databases has long posed a challenge in molecular biology. This issue is particularly acute for viral proteins, which demonstrate limited homology to known proteins when using alignment, k-mer, or profile-based homology search approaches. A novel methodology employing Large Language Models (LLMs) addresses this methodological challenge by annotating protein sequences based on embeddings.

**Results:**

Central to our contribution is the soft alignment algorithm, drawing from traditional protein alignment but leveraging embedding similarity at the amino acid level to bypass the need for conventional scoring matrices. This method not only surpasses pooled embedding-based models in efficiency but also in interpretability, enabling users to easily trace homologous amino acids and delve deeper into the alignments. Far from being a black box, our approach provides transparent, BLAST-like alignment visualizations, combining traditional biological research with AI advancements to elevate protein annotation through embedding-based analysis while ensuring interpretability. Tests using the Virus Orthologous Groups and ViralZone protein databases indicated that the novel soft alignment approach recognized and annotated sequences that both blastp and pooling-based methods, which are commonly used for sequence annotation, failed to detect.

**Conclusion:**

The embeddings approach shows the great potential of LLMs for enhancing protein sequence annotation, especially in viral genomics. These findings present a promising avenue for more efficient and accurate protein function inference in molecular biology.

**Supplementary Information:**

The online version contains supplementary material available at 10.1186/s12859-024-05779-6.

## Introduction

The association between amino acid sequences and corresponding protein function is a long-standing problem in molecular biology [1, 2]. Despite substantial efforts towards addressing this issue, a significant number of public database sequences lack functional annotations [3]. The annotation gap is notably problematic for viruses due to their high mutation rates and vast sequence diversity [4]. In sequence-based protein annotation, amino acid sequences are compared with known protein sequences from public databases using homology-based (e.g. BLAST) or profile-based (e.g. Hmmer) approaches.

Homology-based predictions infer the function of a query sequence based on its best alignment with one or more known functional sequences, utilizing a predetermined substitution matrix that measures amino acid similarity. The resulting alignments can highlight conserved regions thus revealing structural and functional relationships between sequences. Substitution matrices used in judging alignment accuracy, are derived from empirical biochemical data and do not consider the context in which amino acids occur. For instance, when employing the PAM250 substitution matrix, aligning the amino acids asparagine (N) and tryptophan (W) results in a negative alignment score of − 5, regardless of the broader structural context where these amino acids occur. As such, much care and effort has gone into constructing substitution matrices, which assume unbiased amino acid compositions. Currently, all protein database search methods use standardized amino acid substitution matrices for scoring and assessing the statistical significance of sequence alignments [5]. BLAST remains the predominant homology-based tool that leverages substitution matrices for sequence annotation, with its two primary publications cited 102,958 and 82,934 times, as of July 2023 [6, 7], respectively.

Recent studies have also explored the use of sophisticated neural networks to learn to predict protein function [8–10]. The advanced computational capabilities of these networks allow researchers to uncover complex patterns and relationships, enhancing the accuracy and depth of protein function predictions. For instance, the VPF-PLM model uses protein embeddings and a feedforward neural network to categorize input proteins into one of nine predefined PHROG family categories. However, these and similar neural network-based methods face limitations in their adaptability to predefined classes-such as the nine categories in this example. Adapting these methods to new classes necessitates sourcing new training data and the reengineering and retraining of the neural network. Specifically, VPF-PLM employs pooled embeddings, which average data across sequences and may mask regions indicative of distinct characteristics. This averaging out can lead to the oversight of localized sequence features. Furthermore, neural network-based classification methods offer limited insight into their decision-making processes, making them less adaptable than more straightforward database search methods such as BLAST, which provides clear alignments for user interpretation and facilitates easy database updates.

Profile-based annotation prediction identifies protein function by creating a profile from multiple sequence alignments (MSAs). These methods offer higher sensitivity by considering multiple pieces of evidence rather than a single pairwise alignment, capturing expected amino acid variability in a sequence. Despite increased sensitivity, profile-based sequence alignment is challenging because building a reliable MSA for rare sequences can be difficult or even impossible due to the limited availability of similar sequences in databases. Additionally, profile-based alignments are highly dependent on MSA quality, thus errors or biases in the MSA impact the alignment results. Consequently, single-sequence homology-based methods, such as BLAST, remain the de-facto standard in sequence-based protein functional annotation.

Protein–protein interactions represent another class of solutions used in protein function annotation and have been extensively studied [11]. Protein interaction networks provide a framework for understanding the relationships between genes and phenotypes, as well as the mechanistic basis for cellular functions [12]. Early methods for assigning functions to unannotated proteins relied on the frequencies of interaction partners having known functions [13]. More recently, techniques incorporating graph embedding and machine learning have been developed [14, 15]. These modern approaches utilize low-dimensional representations of interaction networks for capturing essential features and patterns in protein interactions, identifying functionally related proteins even in the absence of annotated homologs. However, protein–protein interaction networks are poorly suited for functional annotation of viral proteins because a substantial proportion of existing viral sequence space remains unannotated.

### The large language model revolution

Representation learning is the process through which machine learning algorithms acquire compact and meaningful representations of input text, referred to as embeddings [16]. Proteins and text share a common characteristic as both can be represented as sequences of discrete elements, amino acids for proteins and words for text. Because of these structural similarities, the same representation-learning techniques can be applied to both proteins and text.

Large Language Models (LLMs) are a key foundational technology behind artificial intelligence (AI) and natural language processing (NLP) [17]. LLM-enabled AI systems process and generate text by utilizing deep learning techniques and training on vast quantities of textual data. LLMs have brought about significant advancements in natural language processing, offering numerous advantages and a broad array of potential applications. Development and widespread adoption of LLMs, which significantly improve performance on various tasks such as machine translation and question answering, have driven the revolution in natural language AI. The key innovation in the most recent NLP neural network architecture, transformers, is the self-attention mechanism. Through this mechanism, the model weighs and focuses on different parts of a sequence when encoding a word. For instance, the word “bank” can refer to a financial institution, a strip of land along a river, or even an aviation maneuver, depending on the context. Encoding linguistic features and nuances through context has been crucial to the success of LLMs.

In NLP, embeddings are typically computed at the word level, or token, meaning that each word chunk in the text is mapped to a unique continuous vector representation in a high-dimensional space. As a consequence, semantically similar words are close to each other in the vector space [18]. This word-level analysis captures word relationships revealing the semantic and syntactic meaning of the text. Various language-based approaches have been successfully applied to protein sequences revealing a continuous representation of amino acids in a high-dimensional space, where the amino acids show close proximity in vector space if they have similar functions in the context of the input sequence [19–21]. Embeddings have been used as inputs to various machine learning models for tasks such as protein classification, protein–protein interaction prediction, and protein function prediction [22, 23].

Protein sequence embeddings can be derived using methods utilizing biochemical data (e.g., amino acid physicochemical properties, isoelectric point, hydrophobicity, and polarity) [24] or in an unsupervised manner, such as using bag-of-words [25], n-grams [26], term frequency-inverse document frequency (TF-IDF) [27] or deep neural networks [28, 29]. Building on the success of LLMs, recent developments in protein embedding have utilized transformer-based architectures for deriving high-quality embeddings of protein sequences. These models use self-attention mechanisms that capture complex relationships between amino acids and their 3D structures, thus creating embeddings that represent nuanced information about the sequences [30, 31]. As a result, transformer-derived embeddings have demonstrated significant improvements in accuracy across a range of applications from mutational effect and secondary structure to long-range contact and protein structure prediction [19, 31–35].

Relying on an aggregate representation for a sequence of words, such as sentences and paragraphs, rather than word-level representations is appropriate for many NLP tasks. A number of methods have been proposed for constructing a comprehensive representation based on individual word-level embeddings. A commonly used method involves pooling the individual word representations to generate a unified sequence representation [36, 37]. The resulting pooled representations can then be compared using various similarity measures, such as cosine distance, which calculates the cosine of the angle between two vectors and is frequently used to assess the similarity between embeddings. However, in the context of proteins, we hypothesize that pooling amino acid embeddings into a single protein-specific representation leads to information loss, similar to other compression methods. This loss of information could potentially result in incorrect or incomplete annotations, thereby affecting the accuracy of the analysis.

It is also possible to infer distances between sequences of words without resorting to pooling techniques. An example of this is the Word Mover’s Distance (WMD), which measures the dissimilarity between two paragraphs as the minimum amount of distance that the embedded words of one document need to “travel” to reach the embedded words of another document [38]. This is similar to global alignment methods, which try to identify matching amino acids with the smallest distance, according to the distance matrix used. However, employing WMD in the context of aligning amino acid sequences faces two primary challenges. First, the WMD may lead to word alignments that are not suitable for aligning amino acid sequences, as it could include transversions. Second, WMD computational complexity, denoted by $O(p^3 log p)$, where *p* represents the number of unique words in the document, is computationally intensive. In addressing these challenges, it is crucial to explore alternative methods or adaptations to WMD that can accommodate the specific requirements of amino acid sequence alignment while maintaining computational efficiency.

The adaptation of LLM methods to protein sequence homology detection was tested by developing a computational pipeline incorporating a soft-alignment alignment scoring approach analogous to pairwise alignment scoring in traditional homology search methods. Functional annotation of viral proteins, which is a significant challenge for traditional homology search methods, was used as the test case for the pipeline. The soft alignment algorithm was both computationally tractable and interpretable, using statistics and an approach similar to that of the popular BLAST algorithm. Rigorous testing using data from Virus Orthologous Groups (VOG) [39], and PFAM [40] databases demonstrated that embedding-based alignment scores were more complete and accurate than blastp. These results indicate that the reported soft alignment approach substantially improves the functional annotation of viral protein sequences.

## Methods

The function of an unknown protein sequence was inferred using a three-step process. Instead of distance matrices, embeddings were used for identifying homologous protein sequences. First, embeddings were generated for a query sequence. Second, an embeddings database of subject sequences was searched using a heuristic for the most similar sequences. Finally, soft alignments, an approach conceptually similar to pairwise global sequence alignments, was used to identify homologous residues, and, thus, estimate the similarity between the query sequence and its closest neighbors.

### Generate protein embeddings

Protein sequence embeddings are derived from a pre-trained (ESM2) transformer with 36 layers and 3 billion parameters, and an embedding size of n = 2500 [31]. The ESM2 model was trained on 3.016 M clusters containing approximately 250 M sequences from the UniRef90 dataset [41].

For any query sequence $q \in S$ of length *n*, *q*’s embedding $E(q) \in \mathbb {R}^{n \times 2500}$ is obtained using the function *E*. In essence, the embedding function *E* maps every amino acid, or token, in each query sequence $q \in S$ to a vector of real values in $\mathbb {R}^{2500}$, denoted by $E(q) = (e_{1}, e_{2}, \ldots , e_{2500})$ for each token in the sequence. In order to condense this information into a single vector, a pooled embedding, $P(q) \in \mathbb {R}^{2500}$, is obtained by averaging the embeddings at each position across all *n* tokens. In mathematical terms, each component $p_i$ of the pooled embedding $P(q) = (p_1, p_2, \ldots , p_{2500})$ is computed as:

$$\begin{aligned} p_i =\frac{1}{n} \sum _{j=1}^{e_{ji}} \end{aligned}$$

where $e_{ji}$ is the *i-*th component of the embedding of the *j-*th token in sequence *q*. In other words, $p_i$ is the average of the *i*th component of the embeddings of all *n* tokens in the sequence.

### K-nearest neighbors embeddings search

For a query *q*, let *P*(*q*) be the pooled-embedding vector representing the query *q* as described in Step 2.1. An embedding database was built from a set of annotated subject sequences *S*, where each subject sequence *s* is associated with a unique vector *P*(*s*). The embedding database was implemented using FAISS [42], a specialized library designed for efficient similarity search and clustering of dense vectors. The database is stored as an *IndexFlatL2* index, which allows exact database search. Prior to insertion into the database, embeddings are normalized using the *faiss.normalize_L2* method. Similarly, query sequences are normalized, allowing the conversion of a cosine distance to a maximum inner product search, and ensuring effective retrieval of the most relevant annotated sequences for further analysis and comparison.

The K-Nearest Neighbors (KNN) method [43] retrieves the *k*-nearest neighbors of the query sequence *q* from the embedding database. Specifically, the cosine distance between *P*(*q*) and *P*(*s*) was computed, and the *k* embeddings with the maximum cosine similarity were selected as the *k*-nearest neighbors of *P*(*q*).

### Identify homologous amino acids using soft alignment

For subject sequences identified through KNN search, homologous amino acids between the query and subject sequences were identified in a conceptually similar way to that used for local sequence alignments. Given two protein sequences, a query $q = {q_1, q_2, \dots , q_n}$ and a subject $s={s_1, s_2, \dots , s_m}$, a matrix *D* is defined as the matrix of size $n \times m$, where *n* and *m* are the lengths of protein *q* and *s*. Each value $D_{i,j}$ in the matrix is the cosine similarity between the embeddings (*E*) for amino acids $E(q_i)$ and $E(s_j)$ at positions *i* and *j* in *q* and *s*.

**Fig. 1:**
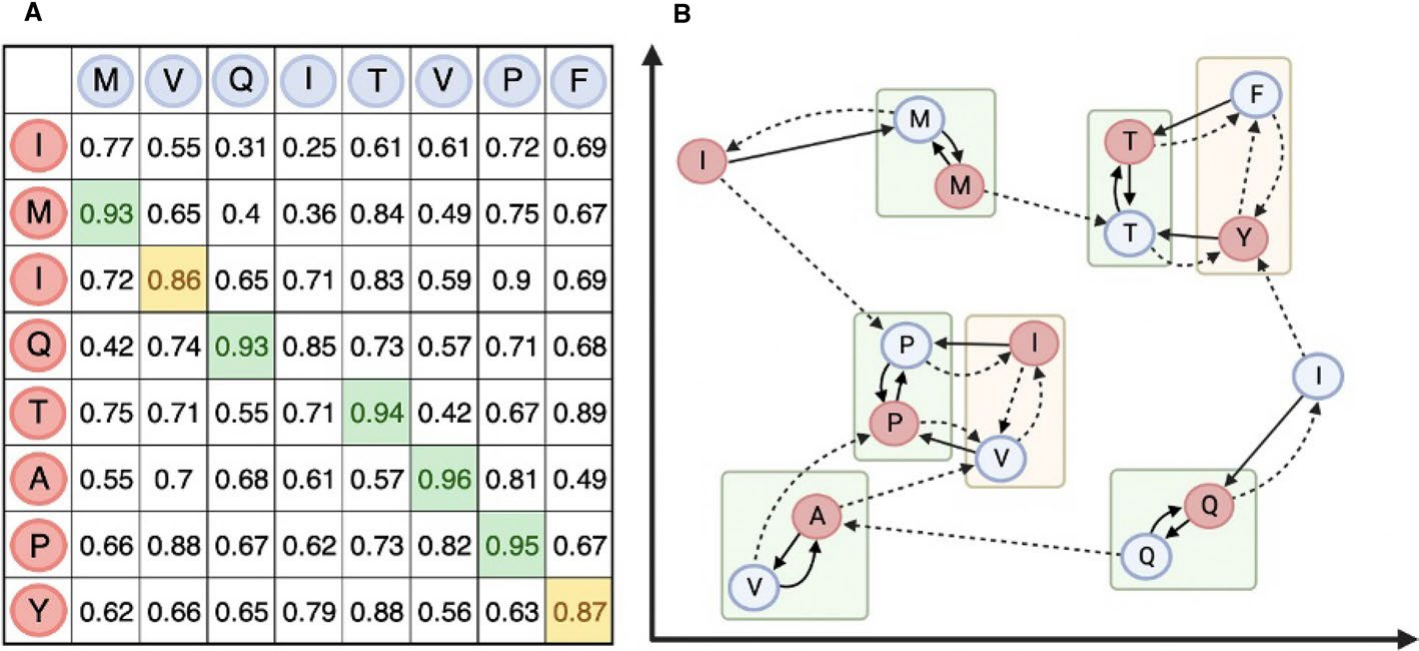
**A** Similarity matrix *D* between a query sequence *q* in red and a subject sequence *s* in blue, both eight amino acids in length. For each amino acid pair, $D_{ij}$ represents the cosine similarity between $E(q_i)$ and $E(s_j)$, the embeddings of the amino acids $q_i$ and $s_j$ respectively. Green cells represent mutual matches, whereas yellow cells are secondary matches. **B** Graph illustration depicting the mutual and secondary matches in *q* and *s*. Each vertex represents an amino acid. Solid arrows link a vertex to its top match, while dashed arrows connect a vertex to its second-best match. The green outline surrounding amino acids indicates mutual matches, where both edges are solid lines. This signifies that the enclosed amino acids are each other’s best match. The yellow outline denotes secondary matches, where at least one of the edges is a dashed line

For each amino acid $q_i \in q$, $C(q_i) = {s_{j_1}, s_{j_2}, s_{j_3}}$ is defined as the top three matching amino acids in *s* with the largest cosine similarity. Similarly, for each amino acid $s_j \in s$, $C(s_j) = {q_{i_1}, q_{i_2}, q_{i_3}}$ is defined as the top three matching amino acids in *q* with the largest cosine similarity. Mutual matches are denoted as pairs of amino acids $(q_i, s_j)$ for which $q_i$ is the highest scoring match in $C(s_j)$ and $s_j$ is the highest scoring match in $C(q_i)$. In other words, $q_i$ is $s_j$ best match and the other way around. Secondary matches are denoted as pairs of amino acids $(q_i, s_j)$ that are not mutual matches but appear in each other’s top three best matches, i.e., $q_i \in C(s_j)$ and $s_j \in C(q_i)$, with the additional condition that neither $q_i$ nor $s_j$ is a mutual match for any other amino acid, i.e., $\forall k \ne i,\ s_j \ne \arg \max _{d \in C(q_k)} \cos (q_k, s)$ and $\forall l \ne j,\ q_i \ne \arg \max _{q \in C(s_l)} \cos (s_l, q)$. This process is illustrated in Fig. 1.

Using a matrix to visualize the mutual and secondary matches for two sequences sharing homology over their embedding distance matrix *D* reveals conserved regions highlighting the degree of sequence similarity. These regions are represented by diagonals, or “alignment paths,” in the matrix between the two sequences. A soft alignment is defined as the path traced through either mutual or secondary correspondences within the matrix *D*, such that the score computed by aggregating the cosine similarities of mutual or secondary matches along the path is optimal.

**Fig. 2:**
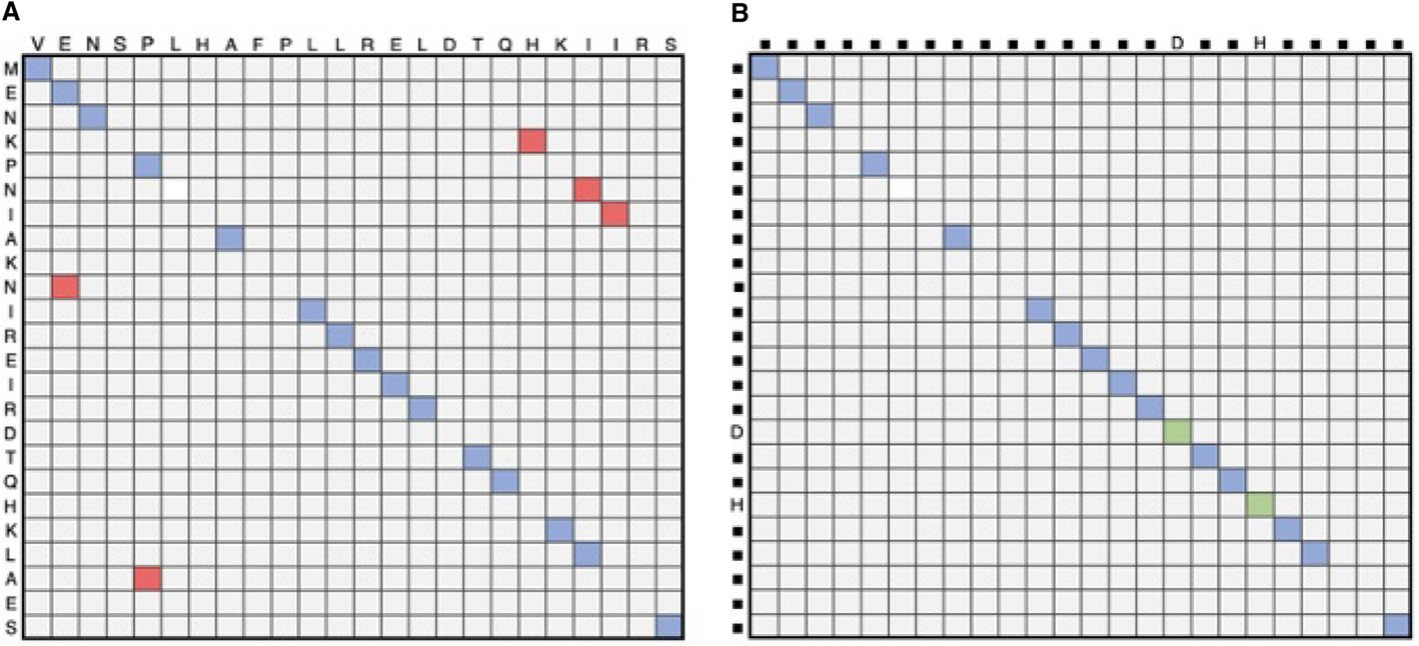
The similarity matrix is traversed removing spurious mutual matches. The soft alignment approach assumes that matches will exist near one another and on a diagonal close to the diagonal representing the alignment. **A** In the first step, the matrix is traversed to identify and discard top-left to bottom-right diagonals with fewer than five mutual matches and which are positioned five insertions or deletions away from a diagonal with more than five mutual matches (cells in red). **B** In the second step, Single gaps along the main diagonal, and representing the same amino acid, are classified as reciprocal matches (green cells), an indication of a false negative

Occasionally, mutual and secondary matches may be misidentified, resulting in matches deviating from the main diagonal. In most cases, off-diagonal matches can be correctly flagged as their inclusion in the alignment path would require insertions in one of the sequences or would require deviating from an otherwise high-quality alignment path to include the mismatch in the alignment. Consequently, prior to computing the embedding-based soft alignment between two sequences, the matrix *D* is first processed to exclude diagonals with fewer than five mutual matches unless positioned within less than five insertions or deletions away from a diagonal with more than five mutual matches (Fig. 2A). Additionally, single gaps on valid diagonals are considered mutual matches if they contain the same amino acid (this is indicated by green cells in the matrix shown in Fig. 2B).

We classify a soft alignment as significant and hence report the pair of involved sequences as homologous providing that the soft alignment score is at least 20. Empirical observations indicated this score most consistently matched a BLAST e-value of 1e−3 using GenBank NR.

## Code availability

A Python implementation of the algorithm described above is available on GitHub (https://github.com/hawaii-bioinformatics/protein_embed_softalign). The accompanying Jupyter notebook presents a comprehensive demonstration of the package. In the default configuration, the code runs on CPU, but it can be changed to use GPU if one is available.

## Experiments

### Experiment 1: Statistical properties of soft alignments in unrelated sequence pairs

The pairwise similarity of one million randomly chosen protein sequence pairs from the ViralZone protein database (https://viralzone.expasy.org/) was analyzed using the WATER tool from the EMBOSS suite [44]. The pairwise similarity was computed as the sum of matches (identities plus positives), normalized by the length of the shorter sequence in each pair. Ten thousand pairs exhibiting the least similarity (maximum of 6% pairwise similarity) were selected, indicating that these sequences might not have been related. These pairs were used as a baseline for determining the occurrence of false mutual matches in unrelated sequences.

### Experiment 2: Annotation into high-level functional classes

The performance of soft alignments, BLAST, and pooled embeddings combined with KNN search, for classifying proteins into broad functional categories was compared. Protein sequences from the Virus Orthologous Groups (VOG) database (http://vogdb.org), which groups NCBI RefSeq viral proteins into protein families using orthology and remote homology, were used as the test dataset. This dataset was annotated with functional categories using a process that combined language-based machine learning to produce annotations from the protein description.

Virus proteins are broadly grouped according to their functions into structural, nonstructural, and regulatory and accessory proteins [45]. In our analysis, we have opted to further categorize structural proteins into capsid and envelope proteins and subdivided nonstructural proteins into those involved in replication or assembly processes. The selected categories were, therefore, (1) Capsid Proteins, (2) Envelope Proteins (3) Replication and Transcription Proteins, (4) Assembly and Release Proteins, and (5) Regulatory and Accessory Proteins (see Table 1). These collective categories provide a detailed breakdown and nuanced understanding of virus protein functions necessary to shed light on the ecological and biological roles of viruses within ecosystems and understand the high-level differences in viral composition across samples, and identify the potential impact of viral infections on microbial host populations.

**Table 1:** The five high-level categories used to classify VOG sequences

Category	Description
Capsid Proteins	Proteins responsible for forming the outer shell, or capsid, of the virus. The capsid provides protection for the viral genome and plays a critical role in viral entry and infection
Envelope Proteins	These proteins are present in the outer envelope of some viruses and are involved in the process of virus entry into host cells
Replication and Transcription Proteins	These proteins are involved in the replication and transcription of the viral genome within host cells, playing a critical role in viral gene expression, replication, and the production of new virions
Assembly and Release Proteins	Proteins playing a variety of roles in the viral life cycle, including regulation of viral gene expression, modulation of host immune responses, and evasion of host defense mechanisms. They are often multifunctional and play critical roles in the survival and spread of the virus as well as maintaining host metabolism during infection
Regulatory and Accessory Proteins	These proteins play a variety of roles in the life cycle of the virus, including regulation of viral gene expression, modulation of host immune responses, and evasion of host defense mechanisms. They are often multi-functional and play critical roles in the survival and spread of the virus within a host organism

The VOG protein database was downloaded on February 20, 2023, from the VOGDB website (http://vogdb.org) and the most current descriptions for VOG proteins were downloaded from the National Center for Biotechnology Information (NCBI). This was necessary as several original VOGDB descriptions did not match NCBI descriptions. For example, protein *YP_004934080.1* had a VOGDB description of a helix-turn-helix transcriptional regulator whereas the NCBI provided a more informative description of CI-like repressor. Given the substantial manual effort needed to validate functional categories, we chose to focus on the 200 most abundant descriptions, cautiously excluding those with ambiguous or incomplete identifiers. This included protein descriptions with keywords such as ‘incomplete’, ‘hypothetical’, or ‘domain-containing’. The corresponding descriptions and their relative abundances are accessible at the following DOI link: https://doi.org/10.5281/zenodo.8206942.

An LLM prompt was engineered to assign protein descriptions to their most fitting functional categories, predicated initially on their roles as inferred solely from these descriptions. This prompt was applied to each protein description for obtaining comprehensive reasoning of the proteins’ roles, derived from the outputs of three LLMs—LLaMa (commit 53011c3 with 65 billion parameters) [46], GPT (v3.5 with 175 billion parameters) [47] (https://platform.openai.com/playground) and Galactica (v1.0.0, large, with 30 billion parameters) [48]. For example, given the input viral protein VP2, LLaMa returned the following: “VP2 is a capsid protein that forms the outer shell, or capsid, of the virus and protects its genetic material. Thus, VP2 is likely a capsid protein.” Similarly, GPT3.5 returned the following: “VP2 is involved in the structure and assembly of the virus, as well as in the interaction with host cells during infection. For example, in parvoviruses, VP2 is a major structural protein that forms the capsid of the virus, while in rotaviruses, VP2 is involved in the attachment of the virus to host cells.” In both cases, the protein was assigned to the “Capsid proteins” category. Sequences not unanimously assigned by all three LLMs or had a probability of less than 0.9 were flagged for manual curation. The probability was computed by normalizing the likelihoods of the top five suggested word completions. The exact prompt used and tables assigning accession numbers to categories are provided in the Additional file 1.

A splitting strategy was used for mitigating potential biases resulting from splitting the VOG dataset into training and test sets, ensuring no orthologous sequences were present in both sets. Specifically, a VOG was randomly selected and its sequences were assigned to the test set. This process was repeated until roughly 20% of the data (13,811 sequences) were assigned to the test set (queries). Subsequently, the remaining 80% of the data (54,886 sequences) were assigned to the training set (subject database). Both the training and test sets consisted of sequences from the same 2134 VOGs, with equal distribution of all five categories in both. This method strengthened the analysis by reducing sequence similarity between the test and the training datasets based on membership in the same VOG. The annotation of each query was based on the functional category of the most similar sequence in the training set. The most similar sequence was identified by three methods: (1) soft alignment based on the highest soft alignment score (minimum score of 20); (2) blastp (v 2.13.0+), e-value $\le$ 1e−3; and, (3) pooled embeddings based on the maximum cosine similarity using the KNN search.

### Experiment 3: Granular functional annotation based on biological processes or molecular functions

Viral proteins from UniProt release 2022_05 corresponding to an annotation score of 3 or 4 (843,999 sequences) were selected for evaluating the efficacy of the soft alignment-based approach for producing granular-level functional assignments. Every protein was associated with at least one UniProt keyword, a standardized vocabulary designed for UniProtKB/Swiss-Prot entries that describes a molecular function or biological process. A total of 35,164 proteins longer than 1024 amino acids (the maximum context size supported by the transformer used to compute embeddings) were discarded. The remaining sequences were dereplicated using CD-HIT (version 4.8.1) with a similarity threshold of 0.9, resulting in 24,070 protein clusters, each annotated with an arithmetic mean of 3.36 molecular functions or biological processes (maximum 9). Annotation of each query was based on the functional category of the most similar sequence in the training set as identified by two methods: (1) soft alignment based on the maximum soft alignment score (minimum score of 20); and, (2) blastp (v 2.13.0+), e-value $\le$ 1e−3.

## Results and discussion

### Results of Experiment 1: Exploring the statistical properties of soft alignments of unrelated sequence pairs

The soft alignments of the 10,000 dissimilar UniProtKB sequence pairs resulted in an average of 1.9 mutual or secondary matches per pair using the criteria of an alignment score of $\ge 20$ and a minimum of $k=3$ consecutive matches, i.e., a gap-free alignment of size 3. The probability of detecting a correspondence between proteins assumed to be non-homologous (less than 6% global similarity) was $\approx$ 1.1e$-$5 for an average amino acid length of 392 in the dataset.

Next, the probability of encountering a gap-free soft-match alignment of size $k=3$ that originated spontaneously was calculated. This is equivalent to observing $k=3$ successive matches extending along the same top-left to bottom-right diagonal in the similarity matrix *D*. It is assumed that $k <=n$, where *n* represents the length of both sequences being aligned.

A similarity matrix *D* of size $n \times n$ contains two diagonals of length 1 (top-right and bottom-left), two diagonals of length 2, two diagonals of length 3, etc$\dots$, and one diagonal of length *n*, referred to as as the “main diagonal.” Thus, a matrix of size $D \in \mathbb {R}^{n \times n}$ has 2 diagonals for each length *i* for $1 \le i < n$, and one diagonal of length *n*.

A gap-free alignment of size *k* can be interpreted as consecutive diagonal elements in the matrix *D*. For each diagonal of length $i \ge k$, there exist $i - k + 1$ sequences of *k* consecutive alignments. Therefore, there are $(n - k + 1)$ potential consecutive diagonal elements of length *k* in the main diagonal, and $2(i - k + 1)$ for $i = k$ to $i = n - 1$ consecutive diagonal elements of length *k* on the remaining diagonals. The total number of *k*-length consecutive diagonal elements, denoted as *S*, is:

$$\begin{aligned} S = 2 \left( \sum _{i=k}^{n-1} i - k\sum _{i=k}^{n-1} 1 + \sum _{i=k}^{n-1} 1 \right) + (n - k + 1) \end{aligned}$$

The probability of picking any *k* consecutive alignments is:

$$\begin{aligned} {n\times n \atopwithdelims ()k}= \frac{(n^2)!}{k!(n^2 - k)!} \end{aligned}$$

Therefore, the probability that *k* randomly selected positions form a consecutive sequence on the same diagonal is:

$$\begin{aligned} P(k| p, n) = S / C(n*n, k) \times p^{k}, \end{aligned}$$

where *p* is the probability of a single match between the two sequences.

Based on the above, the probability of finding k = 3 consecutively aligned amino acids in two protein sequences of $n=400$ amino acids and random single mismatch probability of $p=$1.1e$-$5 and $C(400\times 400, 3) \approx (160000)^3 / (27 \times 4.341))$ is:

1 $$\begin{aligned} \begin{aligned} P(k=3 | p, n)&= 158968 / \text {5.368e+18}((\text {1.1e-5})^3 ) \\&= 3.94\text {e}-29 \end{aligned} \end{aligned}$$

Thus, assuming a single mismatch false positive rate of $p=$1.1e$-$5, which was derived from the compared sequences that exhibit less than 6% similarity, 2.5e+28 pairwise alignments need to be computed to obtain a single alignment that passes the condition of having a gap-free alignment of size 3. Even with a mismatch rate 3 times that which was initially estimated, the probability of having a gap-free alignment of size 3 is still extremely low, indicating the low likelihood of a valid soft alignment arising by chance alone.

### Results of Experiment 2: Annotation results on VOG data

Broad functional annotation was assigned to 13,811 VOG proteins. The likelihood of a trivial function assignment was reduced by ensuring that no VOGs had member sequences in both the test (query) and training sets.

**Fig. 3:**
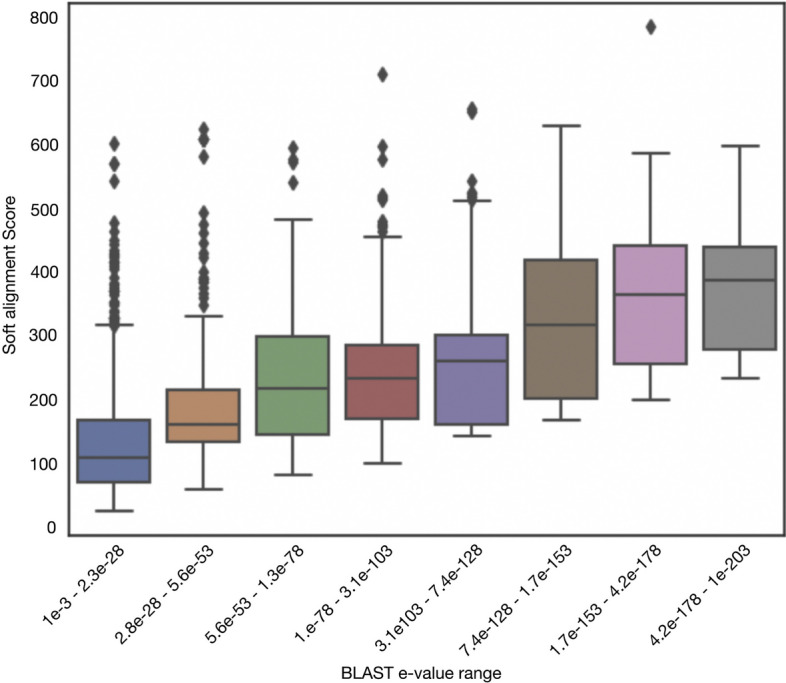
Distribution of the soft alignment scores as a function of the BLAST score. The ranges were chosen to have equal e-value intervals between 1e−3 and 1e−203

The soft alignment approach (minimum soft alignment score 20) annotated a total of 6484 proteins, of which 6107 were correctly annotated (confusion matrix in Table 2a). This outcome yielded a weighted average precision and recall of 0.942 and 0.985, respectively. Conversely, blastp annotated only 2181 proteins, of which 2060 were correctly annotated (Table 2b), resulting in a weighted average precision and recall of 0.944 and 0.986, respectively. It is noteworthy that blastp e-values and the soft alignment score show a statistically significant Spearman correlation of 0.563 (*p* value = 1.12e$-$224) (Fig. 3). This correlation underlines the similarity between the blastp methodology and the soft alignment approach. More significantly, however, the soft alignment approach annotated three times more sequences than blastp using the standard blastp e-value threshold of 1e−3 while yielding similar sensitivity and specificity values.

The pooling-based method (cosine distance threshold of 2.85) annotated 6171 proteins, of which 5176 were correctly annotated (Table 2c), resulting in a weighted average precision and recall of 0.861 and 0.965, respectively.

**Fig. 4:**
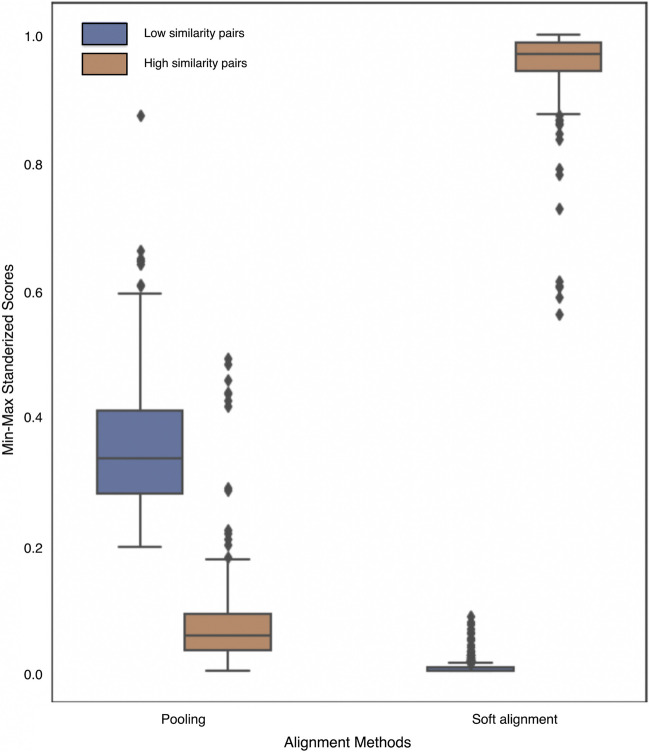
Distribution of min–max normalized pooling distances and soft alignment scores for highly similar (94–99%, blue) and dissimilar (5–10%, orange) sequences. Distances from low- and high-similarity sequences were combined prior to min–max normalization

As illustrated in Fig. 4, the noticeably compact distribution of pairwise distances, predominantly falling between 0 and 4.97 for a large portion of the sequences with only 13 sequences having a distance $\ge$ 5.0 posed a challenge for the pooling method. This narrow range failed to encapsulate the diversity inherent in sequence variability. This was particularly problematic when considering scores at the edges of the similarity range. To illustrate, consider pairs of sequences with low similarity (5–10%) in Fig. 4. For these sequences, pooling distances exhibited considerable variance, with normalized distances ranging between 0.2 and 0.7. Likewise, the distributions of sequences with high similarity (94–99%) also demonstrated substantial variance, with normalized distances varying between 0 and 0.2. Overlap occurred in the distribution of distances of pairs of sequences with low and high similarity, suggesting that pooling could not distinguish between low and high-similarity sequence pairs. In contrast, the distributions of the soft alignment scores between high or low-similarity sequence pairs showed less variance and were substantially separated, indicating a significant difference in similarity between the two groups of sequence pairs (Fig. 4).

**Table 2:** Confusion matrices based on (a) soft alignment-based approach with a soft alignment score threshold of 20, (b) blastp with an e-value cutoff of 1e−3, and (c) pooled embeddings similarity using KNN search for capsid proteins (CP), regulatory and accessory proteins (RAP), envelope proteins (EP), replication and transcription protein (RTP), and assembly and release proteins (ARP)

	CP	RAP	EP	RTP	ARP	Class sensitivity	Class specificity
*(a) Soft alignment (minimum soft alignment score 20)*							
CP	603	0	0	65	15	0.88	0.98
RAP	1	414	0	42	4	0.9	0.99
EP	4	1	83	2	0	0.92	1
RTP	59	59	2	1758	68	0.9	0.98
ARP	13	21	2	19	3249	0.98	0.97
*(b) blastp (e-value cutoff 1e–3)*							
CP	51	0	0	0	7	0.88	1
RAP	0	187	0	53	2	0.77	0.93
EP	1	0	5	0	0	0.83	1
RTP	6	15	0	843	21	0.95	0.95
ARP	2	7	0	7	974	0.98	0.91
*(c) Best match using pooled embeddings*							
CP	675	9	7	80	133	0.75	0.97
RAP	7	435	3	87	76	0.72	0.97
EP	46	6	92	17	8	0.55	0.97
RTP	112	96	11	1680	108	0.84	0.94
ARP	34	66	48	41	3289	0.95	0.94

**Fig. 5:**
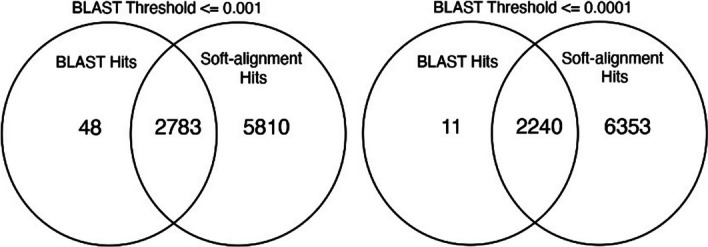
Venn diagrams representing the intersecting and exclusive regions of soft alignments and BLAST results, based on e-value thresholds of 1e−3 (left) and 1e−4 (right)

The soft alignment method failed to detect a few similar sequences that were successfully detected by blastp (Fig. 5). While the KNN step enhances computational efficiency by limiting soft alignment comparisons to five sequences (a parameter that can be changed), this heuristic may yield false negatives due to the limitations of the pooling. Identifying the precise cause of false negatives is challenging. One plausible explanation could be that average pooling accounts for a uniform contribution of each amino acid within the protein. This could diminish the contributions of smaller regions of similarity across proteins, such as short domains, a particular issue for longer amino acid sequences.

False negative/positive outcomes are typical when using heuristics for reducing computational demands. For example, in blastp, an initial “Seed Step” or “Word Finding Step” is utilized for identifying sequences sharing identical matches of a certain length between a query and subject sequences. These matches, often referred to as “k-tuples” or “seeds”, reduce the number of sequences BLAST will consider. However, if the number of these matches is set excessively high, false negatives can occur.

Nevertheless, the soft alignment false negative rate is relatively low, especially when considering the substantial computational savings resulting from using the KNN step. Because the rate of false negatives is contingent on the number of neighbors under consideration, increasing this number could potentially decrease the incidence of false negatives. Alternatively, the process could start with a small number of neighbors, for instance, k = 5, which would filter out sequences with significant matches, and then the unmatched sequences could be re-run using a larger k number, such as k = 20. Testing showed that k = 15 proved sufficient in identifying all 48 hits missed with k = 5 but identified (e-value of 1e−3) (Fig. 5).

**Fig. 6:**
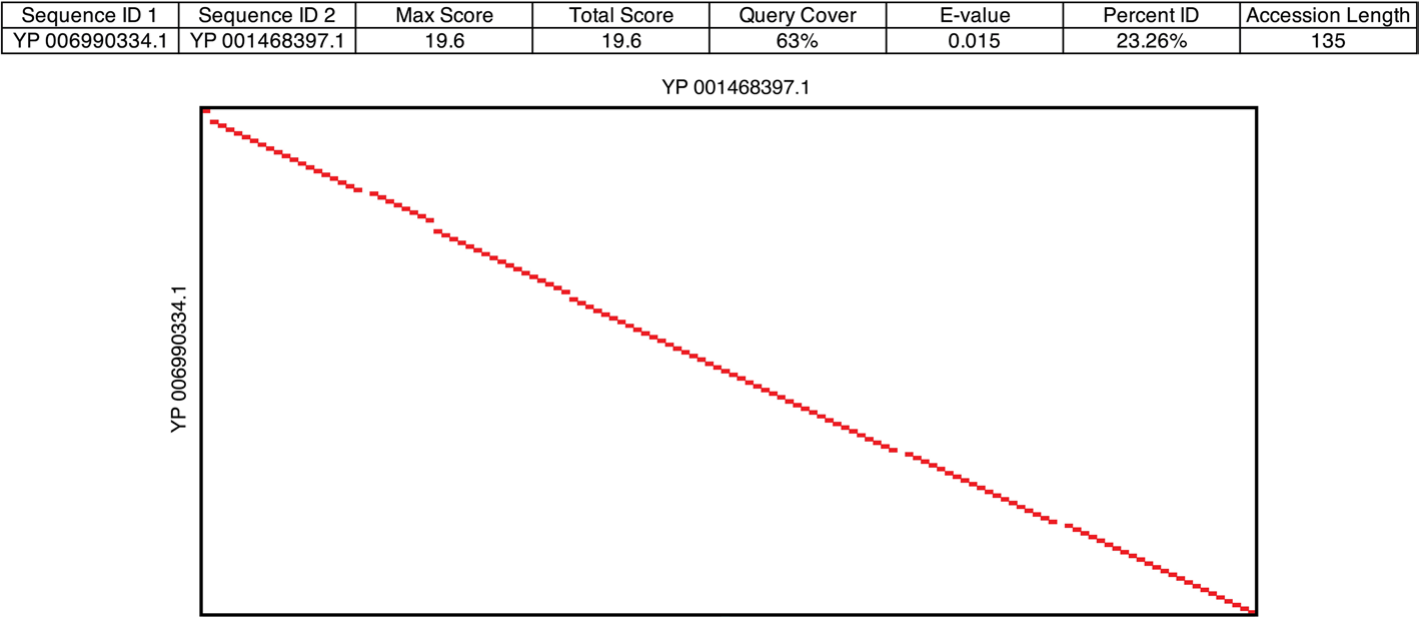
Comparative analysis of sequences *YP_001468397.1* and *YP_006990334*. blastp detected no significant similarity (top table), however a soft alignment found 130 aligned residues between the two proteins of the same length (bottom matrix)

Reliance on a predetermined distance matrix prevents blastp from accepting certain amino acid substitutions in local alignments. For example, two 135 aa long protein sequences, *YP_001468397.1* and *YP_006990334.1*, annotated as Minor head proteins, show no detectable BLAST similarity (e-value of 0.015 and only 23.26% identity) but nearly matched over their complete length using soft alignments (130 out 135 amino acids) (Fig. 6). The reason for BLAST’s inability to identify similarity between the two sequences is detailed in the Additional file 1 (Section II: Analysis of BLAST Results of Minor Capsid Proteins).

Overall, these experiments using non-overlapping test and training VOG peptide databases results show that the soft alignment score is significantly more accurate than the cosine similarity and yields more annotated sequences than blastp. The embeddings-based soft alignment approach correctly assigned function to VOG peptides, allowing rare or novel sequences to be accurately annotated.

### Results of Experiment 3: Assigning sequences into lower-level functional categories

Soft alignments and blastp were used for assigning granular functional annotations to 24,070 dereplicated UniProtKB proteins with known biological processes or molecular function annotations. Soft alignments identified similar sequences (minimum soft alignment score 20) for 16,304 queries, with 16,293 (99%) correctly annotated. In comparison, blastp annotated 16,313 queries (100% correctly annotated).

All 16,293 queries correctly annotated by soft alignments were also correctly annotated by blastp. For the 11 query sequences incorrectly annotated by soft alignments, the discrepancies in top hit ranking occurred predominantly when soft alignment scores for the five KNN search results (step 2 of the algorithm) did not include the best hit identified using blastp. However, false negatives were found to have significant soft alignment scores, i.e., greater than 20, against blastp’s best hits. For example, the KNN step was unsuccessful in identifying *SHUT_ADE02* as one of the top five closest neighbors of sequence *A0A059XDH4_9ADEN*, even though blastp identified it as the top match and despite the presence of 22 mutually matching amino acids spanning 69.5% of the query. Furthermore, nine additional sequences were not annotated by the soft alignment method because alignment scores between each query and its KNN search hits were less than 20, despite the database containing similar sequences. These discrepancies underline the occasional limitations of the pooling method in fully recognizing the intrinsic similarity among sequences. Indeed, while the KNN approach can be a useful tool for reducing the number of sequences a query needs to be compared to, it is important to note that the pooling method is, in essence, a compression method and can potentially distort the true similarity between sequences.

As a solution to protein annotation challenges, our approach emphasizes reducing false positives, under the hypothesis that they can increase the likelihood of errors in downstream analyses. Users can, however, fine-tune the balance between false negatives and false positives by increasing the number of k-nearest neighbors and decreasing the soft alignment score. Such an adjustment can decrease the false negative rate, albeit with the potential trade-off of increasing false positives.

The effectiveness of LLMs is fundamentally connected to the volume and quality of their training data. In domains where training data is scarce, LLMs may generate less accurate embeddings. This issue is particularly evident in sequences that are underrepresented in training datasets, resulting in lower-quality embeddings due to insufficient training on their unique characteristics [49]. Two factors can exacerbate the underrepresentation of proteins in a training swet:

1. *Evolutionary Diversity:* The extensive evolutionary variation among proteins presents a challenge, as the diversity of proteins from lesser-studied species, such as viruses, is often underrepresented in training data.
2. *Function Specificity:* The high specificity of protein functions, which can be affected by minor sequence or structural changes, poses a challenge for LLMs in distinguishing subtle differences, especially in proteins with novel or poorly understood functions.

Despite these challenges, the results presented show that embeddings for viral sequences remain effective for annotating viral sequences. Moreover, it is common practice to fine-tune embeddings by incorporating additional data to cover unrepresented use cases. Therefore, the introduction of superior-quality embeddings would only enhance the alignment quality by enabling the identification of more cross-protein matches. This adaptability ensures that the proposed method remains relevant and effective as new models are introduced, further underlining the significance of our work in the field.

## Conclusion

The protein sequence embeddings followed by soft alignment annotation significantly improved functional annotation of viral proteins with no known homologs. For decades, protein function has been inferred based on the similarity between amino acid sequences. In a general context, viral proteins exhibit significant divergence, leading to challenges in identifying homology. This divergence often hinders the determination of the functional roles of such proteins. When given 13,811 unknown protein sequences, embedding-based soft alignments accurately assigned functions to 5810 more proteins than blastp. In comparison, blastp annotated only 48 proteins that our method missed. Similarly, the meaningful patterns in amino acids detected by our model allowed for the accurate classification of proteins into finer-grained functional categories.

Embeddings derived from specialized language models represent a novel approach to protein sequence annotation. The soft alignment approach was more accurate than cosine similarity, effectively annotating a greater number of previously unannotated sequences than BLAST while maintaining comparable accuracy to blastp for sequencing having database matches. These findings indicate that large language models have the potential to enhance protein sequence annotation significantly, making them a valuable resource for the scientific community studying viruses and particularly for those in viral genomics, which currently lacks annotation for a large number of protein sequences.

### Supplementary Information

**Additional file 1**. This file includes language model prompts used to annotate the protein sequences as well as a brief illustration of the challenges alignment methods like BLAST face when aligning sequences with low amino acids conservation.

## Data Availability

A snapshot of the VOG dataset (vog.faa.tar.gz) was acquired from the official website on February, 2023. The dataset was obtained from the following URL: https://vogdb.org/download. A snapshot of the PFAM Viral Sequences (manually reviewed proteins) was acquired from the ViralZone Website on February, 2023. The dataset was obtained from the following URL: https://viralzone.expasy.org/. The UniProt proteins associated with viral annotations scored higher than 3 or 4 were downloaded from https://viralzone.expasy.org/. The corresponding datasets were downloaded using the subsequent URLs: For annotation score 3: https://www.uniprot.org/uniprotkb?facets=reviewed:false,annotation_score:3 &query=(taxonomy_id:10239), For annotation score 4: https://www.uniprot.org/uniprotkb?facets=reviewed:false,annotation_score:4 &query=(taxonomy_id:10239). For added convenience, this dataset is also accessible on the GitHub repository’s data folder.

## Code availability

*Project name*: Soft Alignment of Protein Embeddings *Project home page*: https://github.com/Hawaii-Bioinformatics/protein_embed_softalign*Operating System*: MacOS or Linux *Programming Language*: Python *Any restrictions for non-academics*: None.
