# Supplementary material for: Improvements in viral gene annotation using large language models and soft alignments
Source: BMC Bioinformatics. 2024 Apr 25;25:165. doi: 10.1186/s12859-024-05779-6 (PMC11046836; doi:10.1186/s12859-024-05779-6)
Supplement: Supplementary file 1 — Additional file 1. This file includes language model prompts used to annotate the protein sequences as well as a brief illustration of the challenges alignment methods like BLAST face when aligning sequences with low amino acids conservation. [file 12859_2024_5779_MOESM1_ESM.pdf]

Supplementary Materials for Improvements in Viral Gene Annotation Using Large Language Models and Soft Alignments

William L. Harrigan Barbra D. Ferrell K. Eric Wommack Shawn W. Polson
Zachary D. Schreiber Mahdi Belcaid

March 15, 2024

I: Language Model Prompt

The following prompt is used to guide the chatbots used for annotating the protein sequences into five primary functional categories.

Classify a viral protein into one of the following categories based on its functions and properties:

- Capsid proteins: These proteins form the virus's outer shell or capsid and protect its genetic material.
- Envelope proteins: These proteins form the outer envelope of some viruses and are involved in virus entry into host cells.
- Replication and transcription proteins: These proteins are involved in the replication and transcription of the viral genome within host cells.
- Assembly and release proteins: These proteins are involved in assembling and releasing new virus particles from host cells.
- Regulatory and accessory proteins: These proteins play a variety of roles in the life cycle of the virus including regulation of viral gene expression, modulation of host immune responses, and evasion of host defense mechanisms.

You should use the chain of thought principle to classify each protein description. Given a protein description, you should first identify the primary role of the protein in the context of viral biology. Using the role, you can predict the protein's class based on its function. If the information provided is insufficient to make a confident classification, the output should be "Unknown."

Examples:
Description: Apoptosis inhibitor IAP.
Role: Apoptosis inhibitor IAP is a protein that can inhibit apoptosis, or programmed cell death, in host cells. In the context of a virus, this protein may play a role in regulating viral gene expression, modulation of host immune responses, or evasion of host defense mechanisms, which are all common functions of regulatory and accessory proteins in the life cycle of a virus.
Class: Regulatory and accessory proteins.

Description: Viral Protein X.
Role: The description of the protein is not provided, so it is impossible to determine its role in viral biology. Further information about the protein's function, domains, and properties would be necessary

to classify it accurately into one of the five categories.
Class: Unknown.

Description: dUTP diphosphatase.
Role: dUTP diphosphatases are enzymes that hydrolyze the nucleotide dUTP to dUMP, which can then be incorporated into DNA. In the context of a virus, a dUTP diphosphatase may play a role in the replication and transcription of the viral genome within host cells. This allows the virus to replicate its genetic material and produce new virus particles.
Class: Replication and transcription proteins.

Description: Zonular occludens toxin.
Role: Zonular occludens toxin is a toxin that can disrupt tight junctions in host cells, leading to changes in the permeability of the host cell membrane. In the context of a virus, this toxin may play a role in the regulation of viral gene expression, modulation of host immune responses, or evasion of host defense mechanisms, which are all common functions of regulatory and accessory proteins in the life cycle of a virus.
Class: Regulatory and accessory proteins.

Description: Uracil-DNA glycosylase.
Role: Uracil-DNA glycosylases are enzymes that can remove uracil residues from DNA by cleaving the N-glycosidic bond between the base and the sugar. In the context of a virus, a uracil-DNA glycosylase may play a role in the replication and transcription of the viral genome within host cells, allowing the virus to replicate its genetic material and produce new virus particles.
Class: Replication and transcription proteins.

Description: {}.

II: Analysis of BLAST Results of Minor Capsid Proteins

The following soft-alignment illustrates the difficulty for standard alignment approaches such as BLAST to align sequences characterized by low conservation of amino acid signatures. Both sequences (YP_001468397.1, YP_006990334.1) are annotated as Minor Capsid Proteins and matched using CD-Search against the protein family representing the Minor Capsid Proteins from bacteriophages, as identified in 33 sequences within the PFAM database entry 12691.

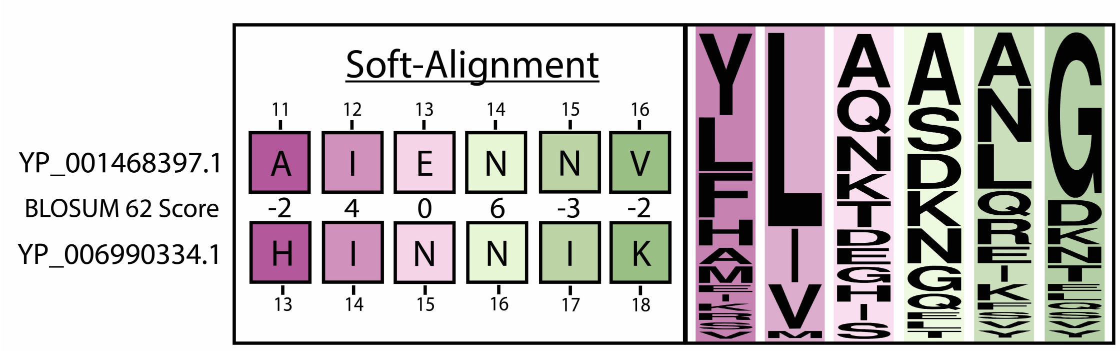

Supplementary Figure 1: Illustration of partial protein sequence soft-alignment and amino acid frequency histogram. On the left, a segment of the PFAM 12691 alignment shows amino acids 11-16 from the protein sequence YP_001468397.1 juxtaposed with amino acids 13-18 from YP_006990334.1. These positions correspond with the first six amino acids of the PFAM12691 multiple sequence alignment. On the right, the histogram represents the frequency of each amino acid at the corresponding positions across all 33 sequences in the PFAM12691 multiple sequence alignment, with the size of the letters indicative of their relative frequency. The color coding of the histogram matches that of the pairwise alignment.

For brevity and clarity, we focus on a small segment—the initial six amino acid-region—of the PFAM alignment (PFAM 12691). The challenge with standard methods, including BLAST, arises from their inability to detect sufficient similarity in instances of uncommonly low match scores, as calculated by commonly used matrices such as BLOSUM. For instance, for this short segment, the total BLOSUM score is only 3, with only one identical amino acid signature aligned. This alignment score is not sufficient to identify homology in this region using BLAST (See Supplementary Figure 1).

Despite the apparent low amino acid conservation rate at the beginning of these sequences, our method successfully identifies this same region of the sequences YP_001468397.1 and YP_006990334.1 as homologous. The proposed soft alignment seems to be in agreement with the multiple sequence alignment (MSA) involving 33 sequences in PFAM 12691. For example, our soft-alignment identifies the isoleucines (I) at positions 12 and 14 as homologous, which match the PFAM MSA, where both amino acids appear in one of the very few highly conserved columns in the MSA. The remaining columns of the soft-alignment are in agreement with the conservation patterns observed in PFAM alignment. For instance, both N and I appear in the fifth column of the PFAM alignment. This example, while simple, underscores the challenge of using BLAST to identify similarities within regions characterized by a single identical amino acid signature match and an exceptionally low total BLOSUM score.
